# Individual differences in circadian locomotor parameters correlate with anxiety- and depression-like behavior

**DOI:** 10.1371/journal.pone.0181375

**Published:** 2017-08-01

**Authors:** Jeffrey Anyan, Michael Verwey, Shimon Amir

**Affiliations:** Center for Studies in Behavioral Neurobiology, Department of Psychology, Concordia University, Montreal, Quebec, Canada; Kent State University, UNITED STATES

## Abstract

Disrupted circadian rhythms are a core feature of mood and anxiety disorders. Circadian rhythms are coordinated by a light-entrainable master clock located in the suprachiasmatic nucleus. Animal models of mood and anxiety disorders often exhibit blunted rhythms in locomotor activity and clock gene expression. Interestingly, the changes in circadian rhythms correlate with mood-related behaviours. Although animal models of depression and anxiety exhibit aberrant circadian rhythms in physiology and behavior, it is possible that the methodology being used to induce the behavioral phenotype (e.g., brain lesions, chronic stress, global gene deletion) affect behavior independently of circadian system. This study investigates the relationship between individual differences in circadian locomotor parameters and mood-related behaviors in healthy rats. The circadian phenotype of male Lewis rats was characterized by analyzing wheel running behavior under standard 12h:12h LD conditions, constant dark, constant light, and rate of re-entrainment to a phase advance. Rats were then tested on a battery of behavioral tests: activity box, restricted feeding, elevated plus maze, forced swim test, and fear conditioning. Under 12h:12h LD conditions, percent of daily activity in the light phase and variability in activity onset were associated with longer latency to immobility in the forced swim test. Variability in onset also correlated positively with anxiety-like behavior in the elevated plus maze. Rate of re-entrainment correlated positively with measures of anxiety in the activity box and elevated plus maze. Lastly, we found that free running period under constant dark was associated with anxiety-like behaviors in the activity box and elevated plus maze. Our results provide a previously uncharacterized relationship between circadian locomotor parameters and mood-related behaviors in healthy rats and provide a basis for future examination into circadian clock functioning and mood.

## Introduction

Disrupted circadian rhythms in physiology and behavior are a core feature of many psychiatric conditions including mood and anxiety disorders [1, 2]. Aberrant sleep/wake cycles (e.g., insomnia, hypersomnia) are observed in various psychiatric conditions [3]. Concurrent to these changes in sleep/wake rhythms, circadian alterations in daily body temperature rhythms, daily activity patterns, and hormone release have also been reported [4, 5]. Because disruptions are observed in several different circadian rhythms, these findings suggest a fundamental connection between the master circadian clock and psychiatric illnesses.

Circadian rhythms in mammals are coordinated by an endogenous circadian clock located in the suprachiasmatic nucleus (SCN) of the anterior hypothalamus. At the molecular level, rhythms in the SCN are driven by a collection of core clock genes that form a transcriptional-translational feedback loop, and go on to orchestrate rhythms in downstream brain regions and the periphery, which produce rhythms in behavior and physiology [6, 7]. Several studies have found links between single nucleotide polymorphisms in core clock genes and mood disorders and schizophrenia [8–12]. Collectively, these findings point to some fundamental circadian mechanism acting at the core of certain affective disorders.

Animal models of depression and anxiety have advanced our understanding of this relationship between circadian rhythms and affective disorders [13]. For example, the unpredictable chronic mild stress (UCMS) paradigm induces a depression-like phenotype in rats that share similar symptoms to humans, such as reduced sexual activity and anhedonia [14]. Remarkably, in this same paradigm, the daily rhythm in the expression of the core clock protein Per2 is blunted in the SCN [15], and the amplitude of this oscillation in clock gene expression correlates with depression-like behaviors [16]. Moreover, mice selectively bred for high anxiety- and depression-like behaviors (HAB) also exhibit aberrant circadian rhythms in the form of fragmented locomotor activity under 12h:12h light-dark (LD) conditions, a longer free running period in constant dark (DD), and are less responsive to the phase shifting effects of light [17]. Treating these HAB mice with the anti-depressant fluoxetine did not alter the free running period but normalized the fragmented rhythms in locomotor activity and light responsiveness (mood-related behaviors were not assessed after fluoxetine treatment) [18]. Finally, it has also been shown that when expression of the core clock gene *Bmal1* is selectively knocked down in the SCN, a behavioral phenotype with increased depression- and anxiety-like behaviors emerged [19]. Therefore, some causal status of SCN-based circadian rhythms can be inferred, and these mouse models of depression and anxiety are demonstrating some essential links between circadian function and certain models of psychopathology.

While there is a clear link between disrupted circadian parameters and mood-related behaviors in animal models of pathology, such links have not been investigated in a healthy population. To fill this gap, several circadian parameters were characterized in a healthy group of rats. We looked at circadian locomotor activity under a 12h:12h LD cycle, during constant darkness, after a 6h phase advance (simulated “jet-lag”), and under constant light. The same rats were then tested on a battery of depression and anxiety-like tests in order to determine which circadian parameters are associated with depression- and anxiety-like behavior. We found several key associations between these circadian parameters and mood related behaviors in this otherwise healthy population of rats.

## Materials and methods

### Animals and housing

Twenty-four inbred male Lewis (LEW/Crl) rats weighing 145-188g upon arrival (Charles River, St-Constant Quebec) were used in this study. To minimize distress to the animals, and provide them with an enriching environment, all rats were individually housed in cages (9.5”x8”x16” deep) equipped with access to running wheels and had ad libitum access to rat chow and water (except for 10-days of restricted feeding, described below). Animals were handled and weighed on weekly basis. At the time of handling, rats were also visually inspected for any health concerns. None of the rats appeared to have health concerns at any point during the study. Each cage was kept in a custom built sound-attenuated and light-sealed ventilated chamber (17.5”x27.5”x27.5” deep). Light cycles were computer-controlled, and programmed to a 12h:12h LD cycle. All procedures were carried out in accordance with the Canadian Council on Animal Care guidelines (http://www.ccac.ca) and were approved by the Animal Care Committee of Concordia University (Montreal, Quebec).

### Lighting schedules

**Fig 1:**
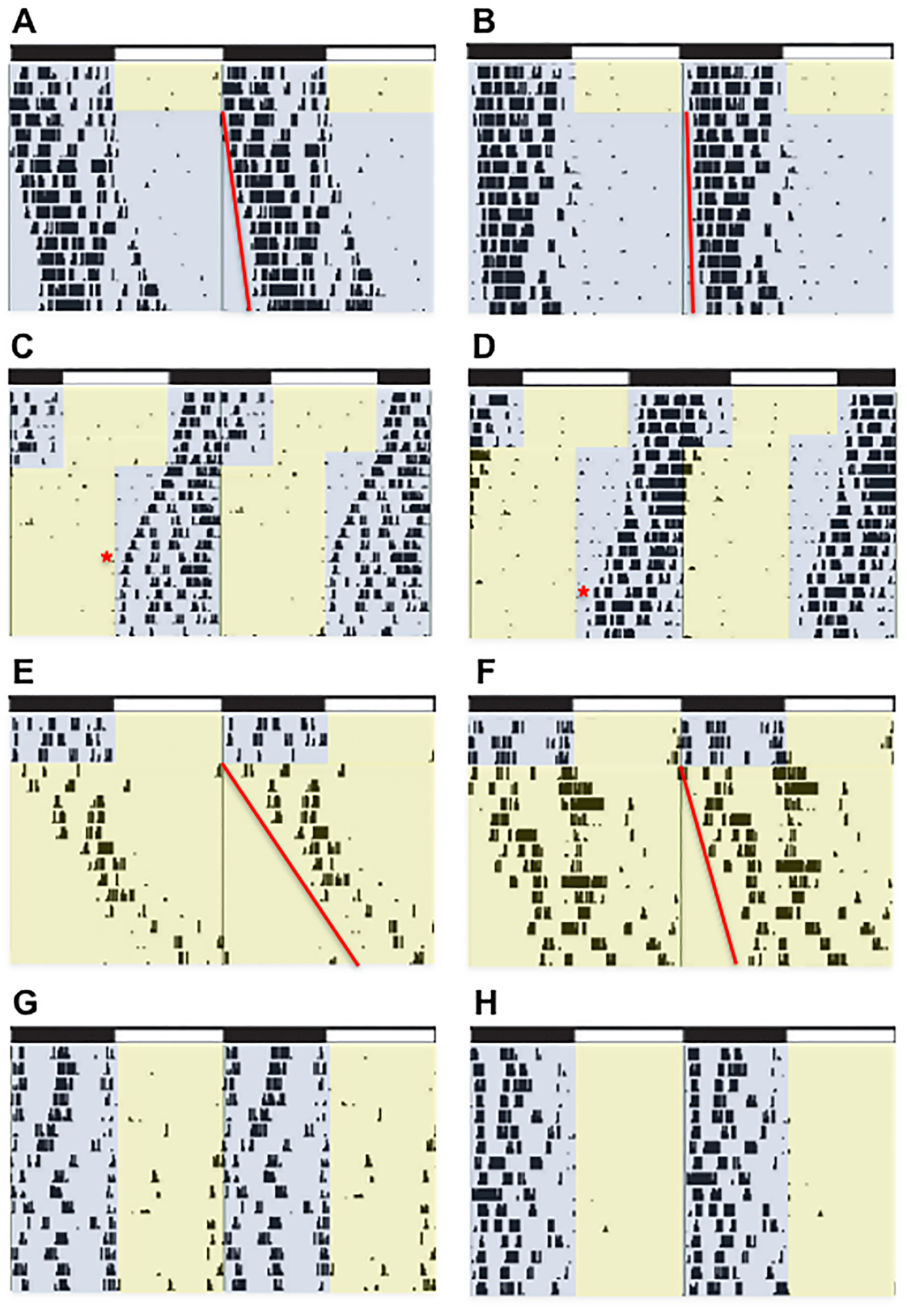
Representative actograms illustrating individual differences under each light each light condition. (A-B) Last 3 days of baseline LD entrainment followed by constant dark (A τ = 24.3, B τ = 24.11.), (C-D) rate of re-entrainment to a 6h phase advance (C = 7 days, D = 11 days), (E-F) 3 days of stable entrainment to 12h:12h LD followed by constant light (E τ = 25.67., F τ = 24.73), and (G-H) entrainment to the final 12h:12h LD after completing all of the light schedules and immediately preceding the start of behavior testing. Fig 1G is representative of a rat with high activity in the light phase and H is a representative of a rat that remains inactive during the light phase.

Rats were entrained to a 12h:12h LD cycle for two weeks, and between each light schedule rats were given another two weeks of 12h:12h LD to re-entrain before subsequent manipulations. All rats underwent the same sequence of manipulations: two weeks of 12h:12h LD, two weeks of constant dark (dark-dark, DD; Fig 1A and 1B), two weeks of 12h:12h LD, a 6h phase advance (Fig 1C and 1D), two weeks of 12h:12h LD, two weeks of constant light (LL; Fig 1E and 1F), and finally were maintained on 12h:12h LD for the behavioral assays of mood and anxiety (Fig 1G and 1H). Under standard circadian notation, zeitgeber time (ZT) 0 denotes the time when environmental lights were turned on, and ZT 12 represents the time when the lights were turned off.

### Circadian analysis of locomotor activity

Wheel running activity was recorded continuously and displayed in 10min bins, and analyzed using VitalView (VitalView software; Mini Mitter Co. Inc., Sunriver, OR). ClockLab (Actimetrics Software, Wilmette, IL) was used to calculate total wheel rotations in the dark, total wheel rotations in the light, activity onset and activity offset over the last 7 days of 12h:12h LD prior to behavior testing. Microsoft Excel (Mac 2011, version 14) was used to calculate percent of activity during the light, variability in activity onset and variability in activity offset using the coefficient of variation equation (Standard deviation/mean*100). Under constant darkness, the free running period was calculated using **χ**^**2**^ over the last 10 days of this 2-week period. Total activity was also calculated for the last 10 days of constant darkness. The rate of re-entrainment to a 6h phase advance was calculated as the number of days required to shift the daily onset of activity to the same phase angle of entrainment observed under baseline. To verify this method, rate of re-entrainment was also calculated by visually inspecting the actograms (with three independent observers). A Cronbach’s alpha of .833 indicates that the two methods are consistent with one another. The free running period and total activity under constant light were calculated in the same way as constant dark.

### Behavioral assays of mood and anxiety

#### Activity box

Each rat was tested in a standard locomotor activity box during the light phase between ZT2-ZT4 (2-4h after environmental lights turned on). Testing was carried out under standard fluorescent lighting because a previous report did not find an effect of illumination on exploratory behaviors or center entries in an open field apparatus [20]. Activity boxes (15”x16.5”x19.5”) with transparent Plexiglas walls and removable plastic tray floors were used, and were cleaned with 70% ethanol between sessions. Each locomotor box was housed in a sound-attenuated chamber and activity was recorded by computer via infrared beam breaks on two sensor rings that created a 16”x16” matrix. Rats were placed in one corner of the activity box and the TruScan Software (TruScan, Activity Monitoring System, Coulborn Instruments Whitehall, PA) collected number of ambulatory movements, total distance travelled (cm), distance travelled in the margins (cm), center time (s). For the purposes of this experiment, ambulatory moves and margin distance are being interpreted as high anxiety-like responses, whereas distance travelled and center time are low anxiety-like responses.

#### Elevated plus-maze

In order to test anxiety-like behavior, a standard elevated plus-maze (EPM) was used. The EPM is an ethologically valid assay for measuring anxiety-like behavior [21]. The maze consisted of two open arms (20”x4”), two closed arms (20”x4”, surrounded by 20.5” walls) and a center square measuring 4”x4”, and the apparatus was 20.5” off the floor. Animals were tested between ZT2-4 under dim red light because it has previously been shown that although circadian phase and illumination do not affect anxiety-like behaviors on the EPM, testing in low light promotes activity [20]. At the start of each trial, each rat was placed in the center square facing one of the open arms and was given 5 min to explore. The maze was cleaned between sessions with alcohol wipes and all trials were video recorded from a camera attached to the ceiling. Videos were scored for percent of open arm entries, time in open arm (s), time in closed arm (s), and time in the center square (s). Percent of open arm entries and time spent in the open arms are low anxiety-like behaviors, more time in closed arms reflects high anxiety-like behavior, and time spent in the center square is interpreted as ‘decision-making’ [22].

#### Forced swim test

A standard depression-related test that is commonly used is the forced swim test (FST) [23], and was administered 3–5 days after the EPM. Testing was performed at the start of the active phase (ZT13-ZT14) under dim red light. Rats were tested under dim red light to prevent exposure to light during the active phase and because it is less stressful for the animals [24]. In this test, glass cylinders (25.5”x10.75” diameter) were filled with tap water (25±2°C) to a depth of 14”. The water was replaced between each test. Rats were habituated to the FST for 15 min on the first day and were tested 24h later for 5 min. Both the habituation and test day were carried out under dim red light and video recorded. After the test, each rat was dried and placed back in its home cage. None of the animals drowned or struggled to survive during the FST beyond what is normal escape oriented behaviors, therefore no experimenter interventions were required. Latency to immobility (s) is being used as a measure of active coping, time spent immobile (s) is being interpreted as passive coping, swimming (s) and climbing are both being interpreted as active coping responses.

#### Fear conditioning

In order to induce contextual fear conditioning, rats were placed in a novel environment (14”x10”x10”) equipped with a stainless steel grid floor connected to a shock source and scrambler (Med Associates Inc. Georgia, VT, U.S.A). On day 1 the rat was placed in the fear conditioning box, and after 3 min of habituation to this novel environment, an aversive foot shock (0.5 mA current for 1s) was delivered, and given again 1 min later. The rat was then removed from the conditioning chamber and returned to the home cage (~30s after last shock). Boxes were cleaned after every test. The next day, each rat was placed back in the same context and the contextual fear response was scored. All testing was conducted at ZT 2 and video recorded for the following conditioned fear responses: latency to freeze (s) and time spent freezing (s).

### Data screening and statistical analyses

All variables were initially screened for outliers. A z-score +/-3 was used as the cutoff and the next closest data point was used to replace the outlier value. All variables were assessed for normal distribution using D’Agostino’s K-Squared test (Prism 6). Scatterplots were visually inspected for linearity, and bivariate correlations were conducted to examine co-linearity. After visual inspection of all relevant scatterplots it was determined that one variable was non-linear (percent of activity in the light). An inverse transformation was applied to produce a linear relationship. A Pearson r = 1.00 between raw and transformed data indicates that the integrity of the data and the distribution were retained after applying this transformation. After data screening was complete, SPSS (version 21) was used to conduct bivariate correlations between behavioral assays and locomotor parameters.

## Results

### Individual differences in locomotor parameters

**Fig 2:**
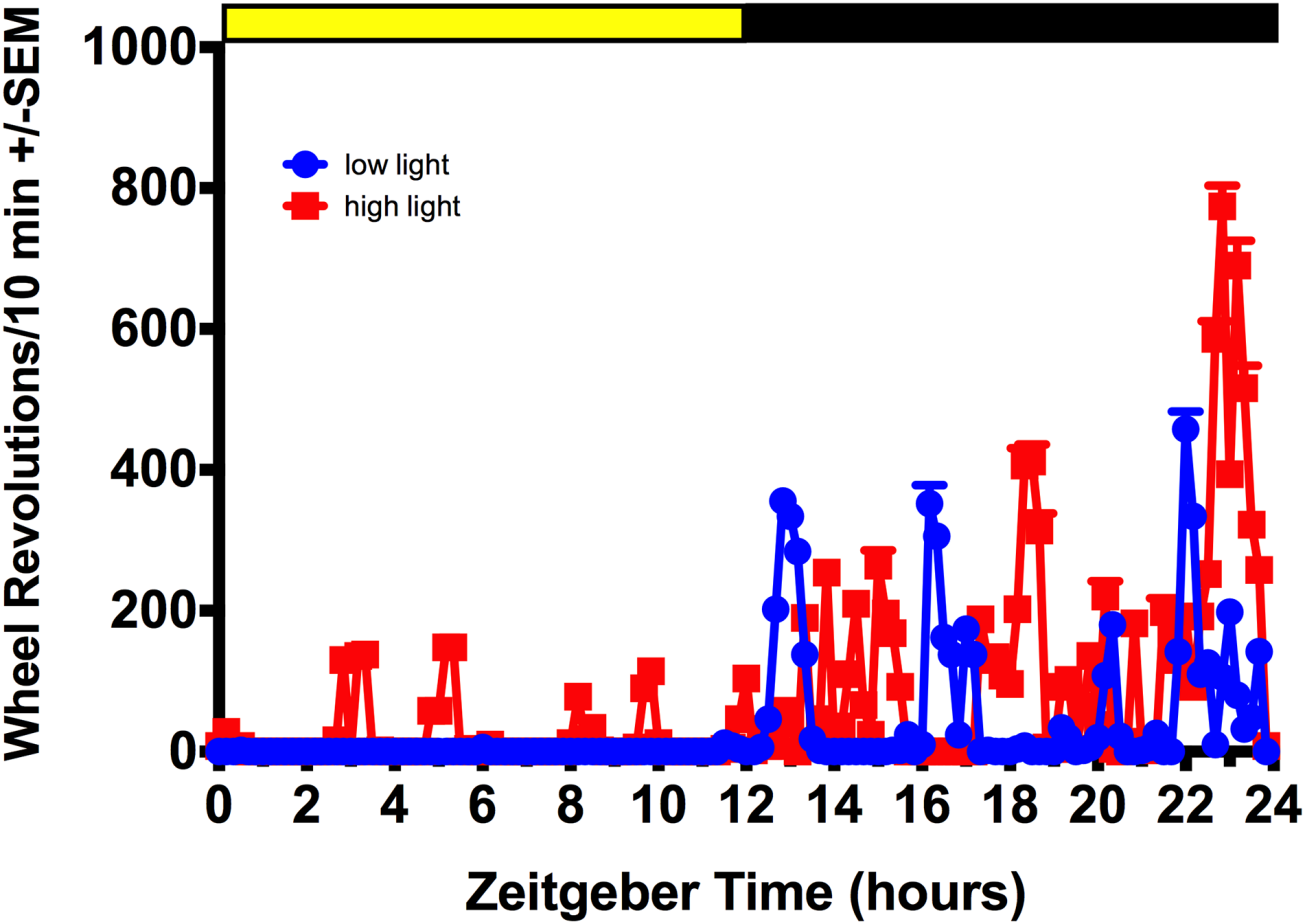
Activity patterns under 12h:12h LD. Representative waveforms of individual variability in circadian locomotor output under 12h:12h LD.

**Fig 3:**
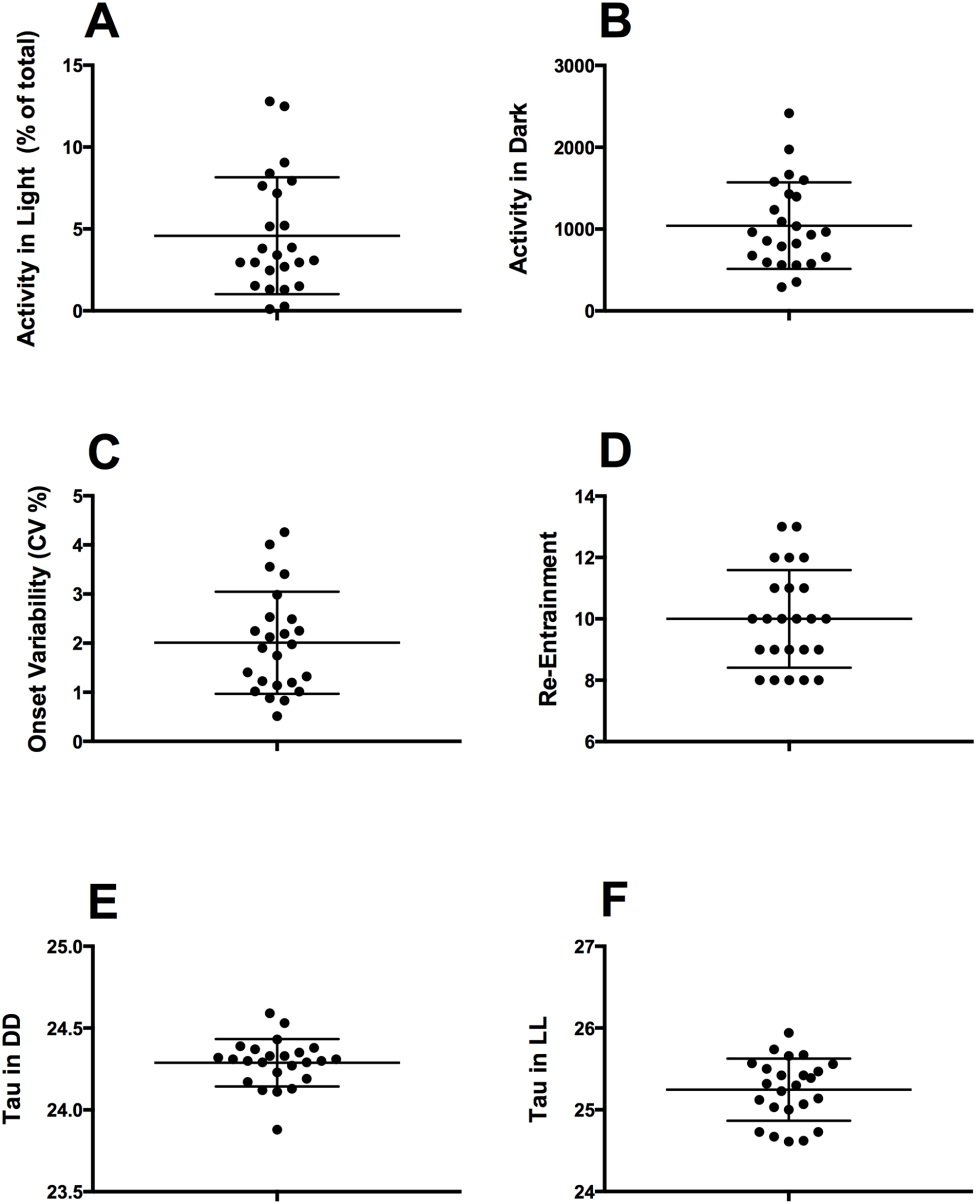
Scatter plot of individual differences in core circadian parameters. (A) Individual variability in total activity in light phase (B) Individual variability in total activity in the dark phase (C) Individual variability in activity onset (D) Individual variability in rate of re-entrainment (E) Individual variability free running period in DD (F) Individual variability free running period in LL.

All behavior testing was carried out during the inactive/light phase except for the FST, which was performed at the beginning of the active/dark phase. On the days where behavior testing was conducted during the light phase, all rats used the running wheel after being placed back in their home cages. This activity was brief and subsided within 1h; 48h post behavior testing all animals have returned to baseline activity in the light phase. See Fig 2 for representative waveforms of daily activity rhythms under baseline conditions and Fig 3 for scatter plots of individual variability in the primary circadian locomotor variables.

### Correlations between locomotor parameters and behavior testing

See Table 1 for complete bivariate correlation matrix between circadian locomotor parameters and mood-related behavior tests.

**Table 1:** Correlation matrix: Circadian locomotor parameters by behavior tests.

		Act. Box				EPM				FST					FC	
		Ambulatory Moves	Total Distance Travelled	Margin Distance	Center Time	% Open Arm Entries	Time in Open Arms (s)	Time in Closed Arms (s)	Center Time (s)	Latency to Immobility (s)	Immobility (s)	Swimming (s)	Climbing (s)	Dives	Latency to Freeze (s)	Freezing (s)
**12:12 LD**	Total Dark Activity	.056	.053	-.165	.260	.011	.249	-.306	.164	-.163	.301	-.194	-.306	-.130	-.004	-.101
	% Activity in Light	.147	-.273	-.015	-.242	-.274	-.309	.332	-.109	.487**	-.362*	.229	.341	-.135	-.035	.116
	Onset Variability	.087	-.038	.210	-.228	-.192	-.372*	.115	.415*	.422*	-.272	.115	.246	.293	-.276	.138
	Offset Variability	.087	.059	.177	-.036	.193	.222	-.182	-.039	.162	-.050	.108	.032	.119	-.279	.085
**Phase Advance**	Re-Entrain	.232	-.136	.454*	-.521**	-.461*	-.394*	.401*	-.098	.334	-.312	.132	.314	-.131	-.081	-.178
**Constant Dark**	Period in DD	.318	-.466*	-.510**	.015	.077	-.030	.006	.045	.021	.038	-.187	-.023	.039	.029	.187
	Activity: DD	.018	-.137	-.272	.164	-.088	-.093	.154	-.133	.044	.172	-.089	-.172	-.438*	.134	.062
**Constant Light**	Period in LL	-.201	-.095	-.031	.084	-.008	.079	-.105	.074	-.112	.245	-.113	-.269	-.307	.298	-.197
	Activity: LL	.321	-.315	-.196	-.063	-.056	-.031	.174	-.292	.101	-.004	-.197	.029	-.193	-.243	.205

Act. Box, activity box; EPM, elevated plus maze; FST, forced swim test; FC, fear conditioning.

*p <.05,

**p <.01

#### Total activity under 12h:12h LD

**Fig 4:**
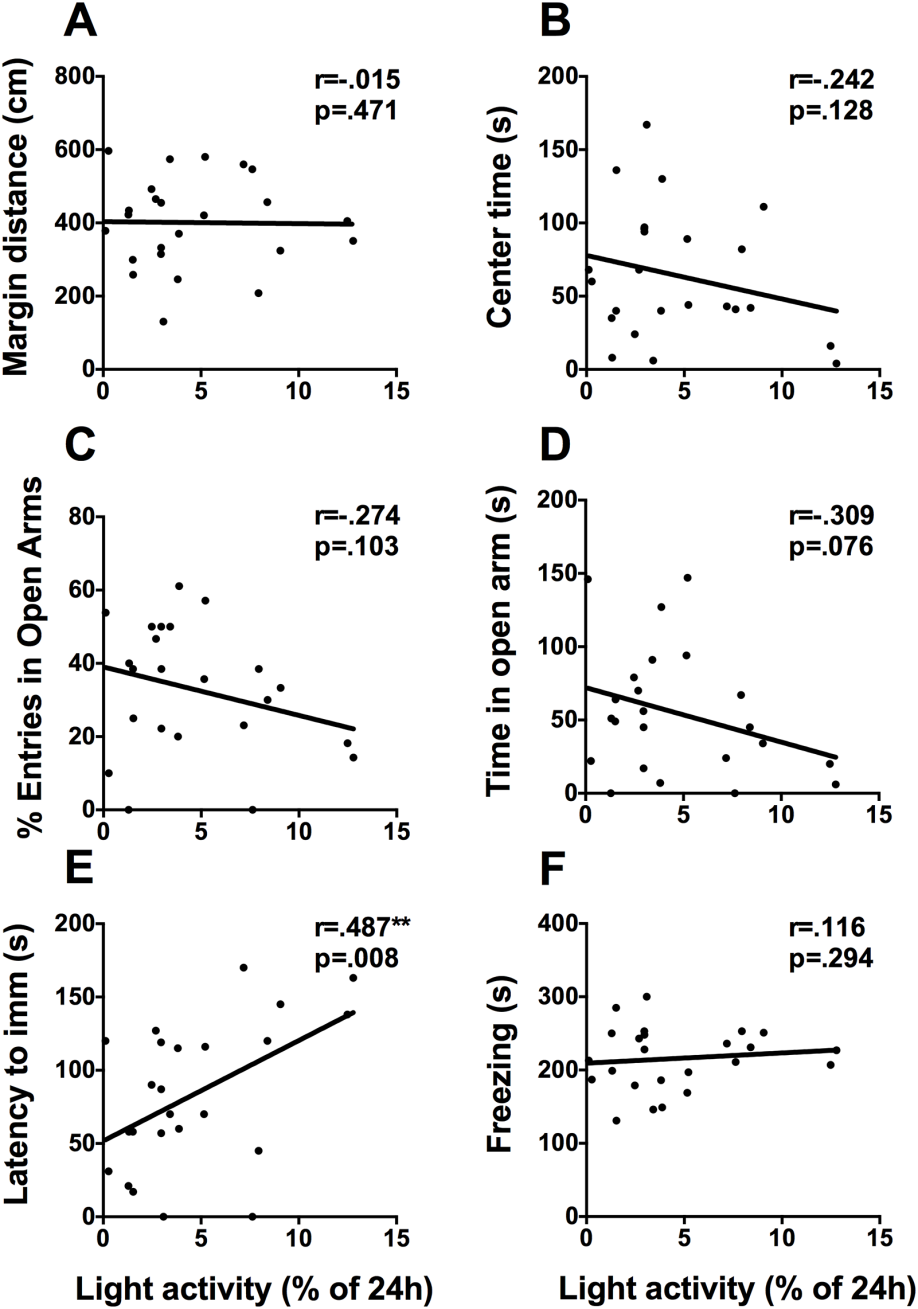
Activity in the light phase is positively correlated with latency to immobility in the FST. Scatterplots representing the relationships between percent of activity in the light phase and (A) distance travelled in the margins of the activity box, (B) time spent in the center of the activity box, (C) percent of open arm entries, (D) time spent in the open arms of the EPM, (E) latency to immobility in the FST, and (F) time spent freezing on day 2 of contextual fear conditioning. * p <.05, ** p <.01.

To examine the relationship between circadian locomotor activity under stable entrainment to a 12h:12h LD cycle we correlated activity in the dark phase and light phase with behavioral assays of mood and anxiety. Total activity in the dark phase did not correlate with any mood-related measures. To assess individual differences in the masking effect of light, percent of activity in the light phase was correlated with each behavioral assay (Fig 4). Percent of activity in the light phase correlates positively with latency to immobility in the FST (r = .487, p = .008. Fig 4E) and negatively with time spent immobile in the FST (r = .-362, p = .041) but not with any of the anxiety measures.

#### Variability in activity onset and offset under 12h:12h LD

**Fig 5:**
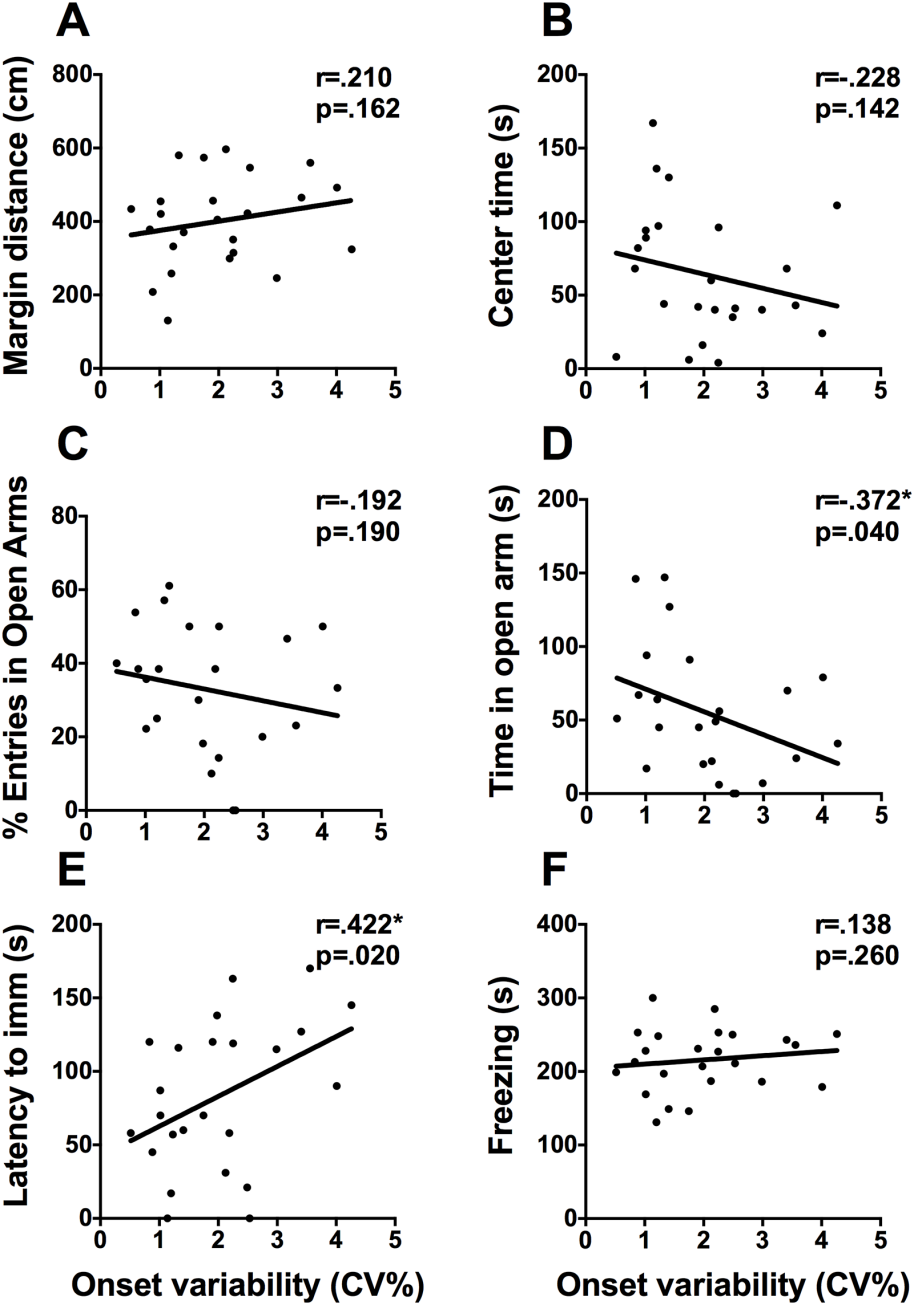
Variability in onset is associated with performance on the EPM and FST. Scatterplots representing the relationships between variability in onset (CV) and (A) distance travelled in the margins of the activity box, (B) time spent in the center of the activity box, (C) percent of open arm entries, (D) time spent in the open arms of the EPM, (E) latency to immobility in the FST, and (F) time spent freezing on day 2 of contextual fear conditioning. * p <.05, ** p <.01.

Variability in activity onset and offset was calculated as a proxy for the precision of the SCN-based clock in each animal [25]. Scatterplots for variability in activity onset by core mood-related measures are shown in Fig 5. Variability in onset correlates positively with latency to immobility in the FST (r = .422, p = .020. Fig 5E), time spent in the center square of the EPM (r = .415, p = .024), and correlates negatively with time spent in the open arms of the EPM (r = -.372, p = .040. Fig 5D). Variability in activity offset was not associated with any of the behavioral assays, p>.05.

#### Rate of re-entrainment

**Fig 6:**
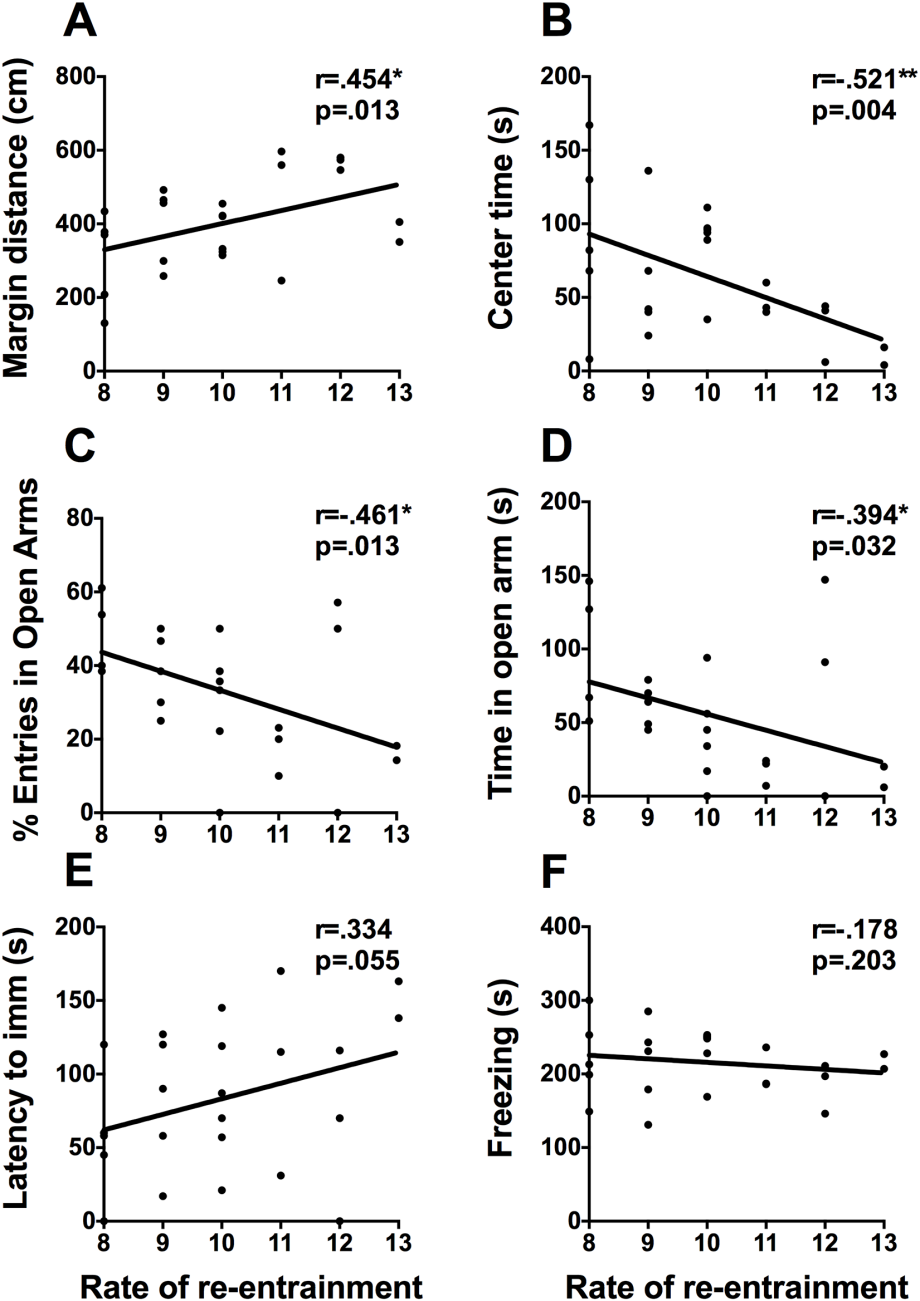
Rate of re-entrainment and mood-related behaviors. Scatterplots representing the relationships between rate of re-entrainment to a 6h phase advance and (A) distance travelled in the margins of the activity box, (B) time spent in the center of the activity box, (C) percent of open arm entries, (D) time spent in the open arms of the EPM, (E) latency to immobility in the FST, and (F) time spent freezing on day 2 of contextual fear conditioning. * p <.05, ** p <.01.

The number of days required to re-entrain to a 6h advance of the light cycle, a marker of the adaptability of the circadian clock, is associated with performance in the activity box, and the EPM (Fig 6). Rate of re-entrainment correlates positively with distance traveled in the margins of the activity box (r = .454, p = .013. Fig 6A), negatively with time spent in the center of the activity box (r = -.521, p = .004. Fig 6B), percent of open arm entries (r = -.461, p = .013), and time spent in the open (r = -.394, p = .032. Fig 6D) and time spent in closed arms of the EPM (r = .401, p = .029). Re-entrainment is not statistically correlated with any measure from contextual fear conditioning or the FST, however there was a trend towards significance with latency to immobility in the FST (r = .334, p = .055, Fig 6E).

#### Constant dark

**Fig 7:**
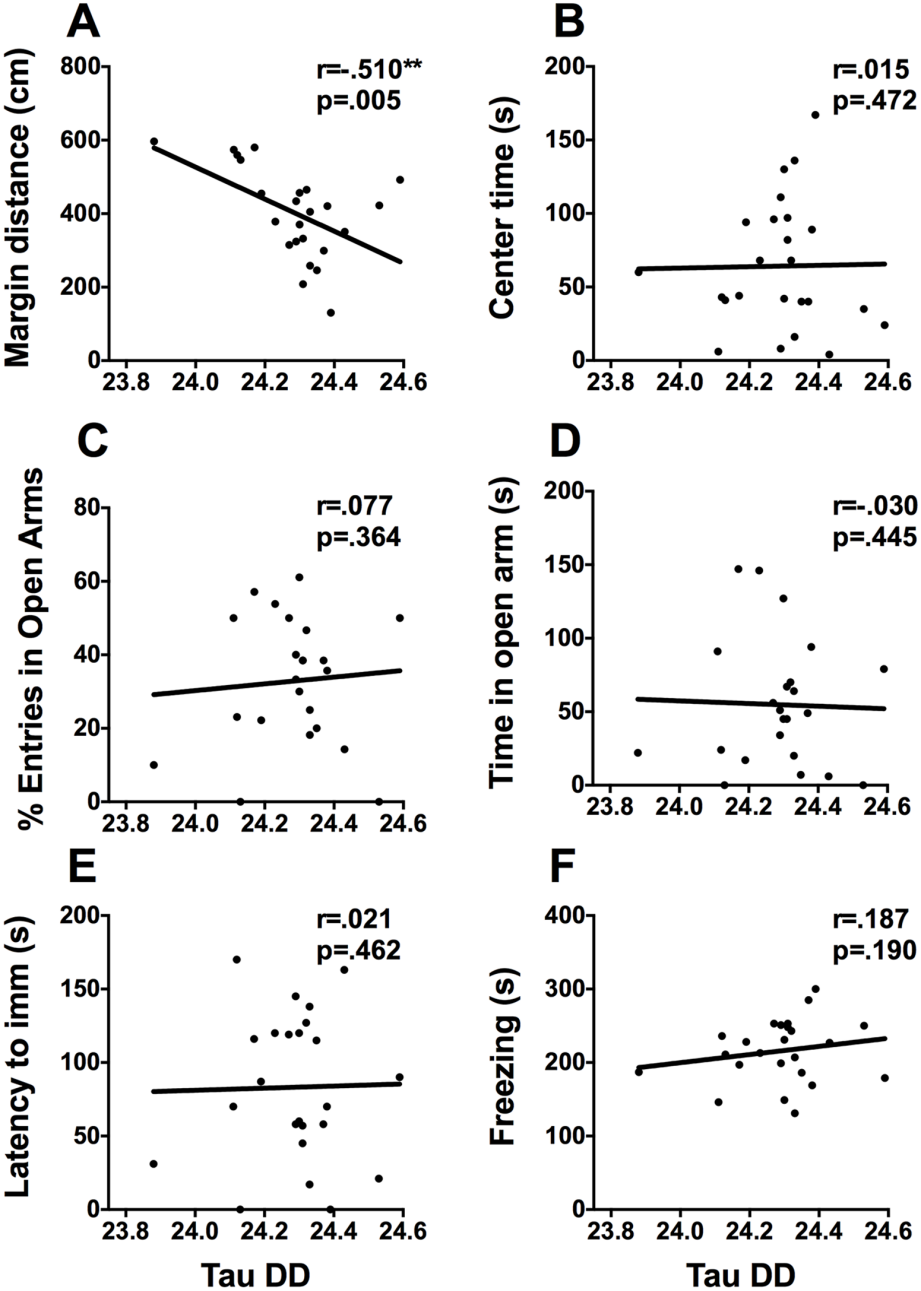
Free-running period in DD and mood-related behaviors. Scatterplots representing the relationships between free running period (tau) in constant dark and (A) distance travelled in the margins of the activity box, (B) time spent in the center of the activity box, (C) percent of open arm entries, (D) time spent in the open arms of the EPM, (E) latency to immobility in the FST, and (F) time spent freezing on day 2 of contextual fear conditioning. * p <.05, ** p <.01.

Free running period under DD correlates negatively with total distance travelled (r = -.466, p = .011) and margin distance in the activity box (r = -510, p = .005, Fig 7A) and correlates positively with closed arm entries (r = .479, p = .01). It does not correlate with any behaviors from the FST (Fig 7 D and 7E). Total activity in constant dark correlates negatively with diving in the FST (r = -.438, p = .016).

#### Constant light

**Fig 8:**
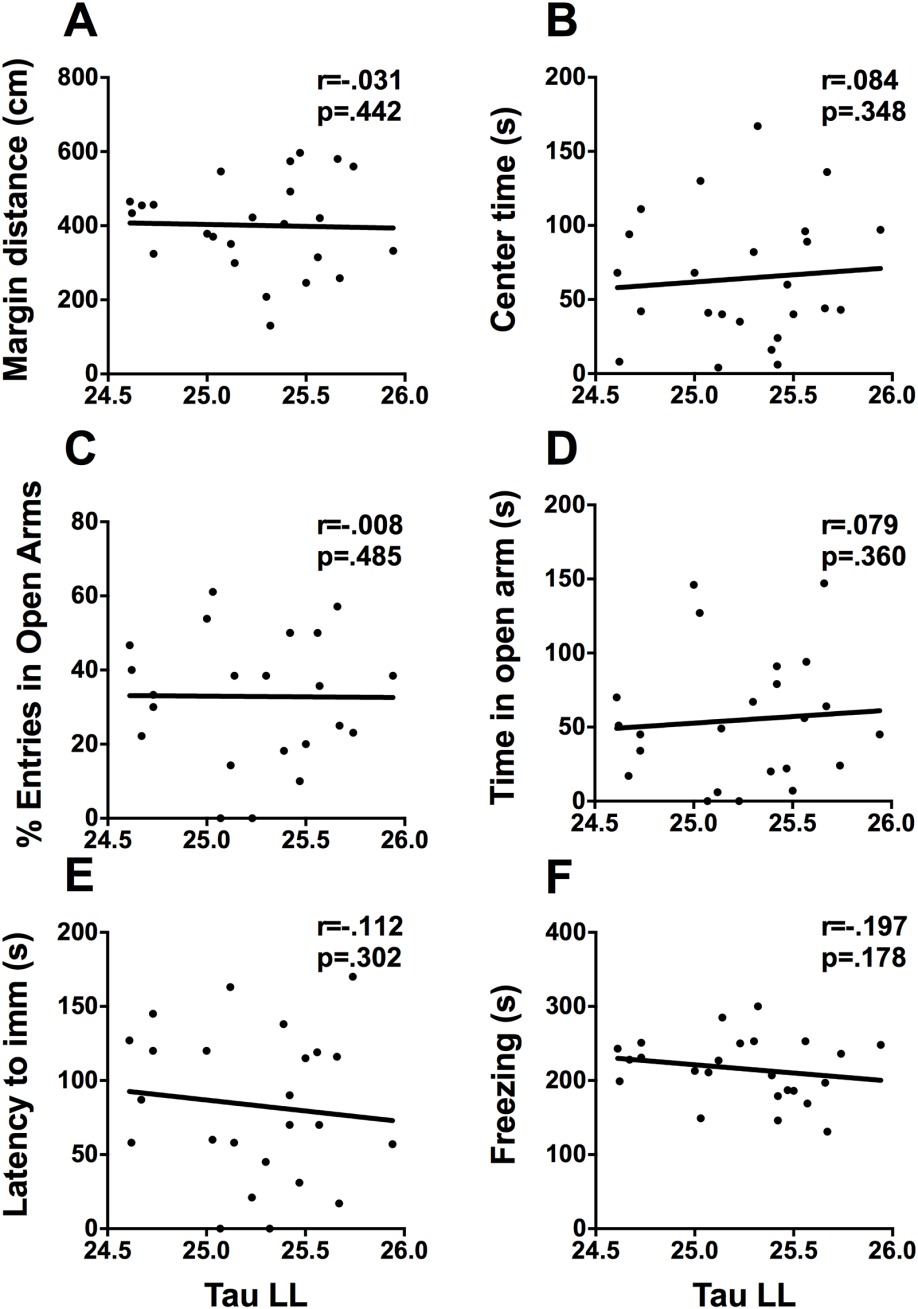
Free-running period in LL and mood-related behaviors. Scatterplots representing the relationships between free running period (tau) in constant light and (A) distance travelled in the margins of the activity box, (B) time spent in the center of the activity box, (C) percent of open arm entries, (D) time spent in the open arms of the EPM, (E) latency to immobility in the FST, and (F) time spent freezing on day 2 of contextual fear conditioning. * p <.05, ** p <.01.

All animals had a measurable free running period under constant light. Neither free-running period nor total activity under LL correlated with any of mood-related measures (Fig 8).

## Discussion

Disrupted circadian rhythms in physiology and behavior are a core feature of mood and anxiety disorders [1, 2]. There is a complex and bidirectional relationship between disrupted circadian rhythms and mood. Numerous studies have shown that environmental (e.g., stress, shiftwork, transmeridian travel) and genetic factors (e.g., clock gene polymorphisms) influence circadian rhythms and are potential risk factors for the development of pathology [15, 26–28]. While there is clearly a role for environmental and genetic factors in the pathogenesis of mood and anxiety disorders, there is also evidence to suggest that aberrant circadian rhythms can be a risk factor for depression and anxiety independently. In the present study we investigated individual differences in circadian locomotor activity and how they relate to depression- and anxiety-like behaviors in a healthy population of inbred Lewis rats. A major benefit of using this inbred strain is that we have previously carried out an extensive characterization of the daily expression clock gene rhythms in this strain [29] and they are commonly used for experiments on stress, mood, and anxiety [30–33]. In the current project, we observed several important associations between individual differences in circadian parameters, based on locomotor activity rhythms, and performance on common assays of depression- and anxiety-like behaviors.

Rats that were more active during the light phase in the home cage under 12h:12h LD, generally had a longer latency to immobility and spent less time immobile in the FST. The FST occurred across 2 days and rats transitioned from being highly active on day 1 to primarily immobile on day 2. Immobility measured on day 2 is frequently interpreted as a sign of ‘despair’, however, it has recently been argued that immobility reflects a switch from active to passive coping [34]. Immobility in the FST may in fact be an adaptive response that promotes survival because rats that become immobile float, conserve energy and are less likely to sink [35]. As a result, the current findings suggest that activity during the light phase is associated with the persistence of active coping in the FST. Increased activity during the light phase could suggest that some rats are less well entrained or are less responsive to the masking effects of light under LD conditions. Therefore, this behavioral result could reflect more general changes in the light-responsivity or entrainment parameters of individual rats.

To evaluate whether entrainment parameters may be associated with behavioral assays of mood and anxiety we also assessed variability in the daily onset of locomotor activity. Similar to the amount of activity during the light phase, higher variability in activity onset was associated with longer latency to immobility in the FST. Variability in onset was also associated with less exploratory behavior and more anxiety-like behaviors in the EPM. Greater variability in onset was associated with more time spent in the center square and less time in the open arms. The present findings demonstrate that variability in onset is associated with active coping in the FST and increased anxiety-like behavior in the EPM. Different factors can account for variability in the precision of activity rhythms, including precision of the SCN clock, the strength of the entraining stimulus as well as individual differences in sensitivity to the entraining stimuli [36]. Because our lighting was standardized, these behavioral results likely reflect individual differences in precision of the SCN clock or individual differences in light responsiveness.

To test the “strength” of entraining stimuli, we measured how long it took rats to adapt to simulated jet-lag in the form of a 6h phase advance of the light cycle. We found that the rate of re-entrainment was associated with anxiety-like behaviors. Rats that took longer to re-entrain spent less time in the center of the activity box but were more active in the margins. Both avoidance of the center square and increased activity in the margins are interpreted as increased anxiety-like response. We observed that rate of re-entrainment was also associated with more time spent in the closed arms of the EPM, a lower percentage of open arm entries, and less time spent in the open arms, which are also indicative of anxiety. It was recently demonstrated that individual differences in the rate of re-entrainment is associated with the degree of phase heterogeneity between individual SCN neurons [37]. These data suggest, that the phase heterogeneity or coherence of neuronal oscillations within the SCN could modulate anxiety-related behaviors.

To explore the relationship between locomotor activity under constant conditions and mood-related behaviors, animals were housed in DD and LL. Although some animal models of depression do exhibit alterations in free running period under DD [38] we did not find any relationship between period in DD and the FST. We did however find an association with anxiety-like behaviours in the activity box and EPM. In respect to constant light we found that total activity in is associated with fewer arm changes in the EPM, suggesting that activity during constant light is associated with less exploratory behavior in the EPM.

We found a strong relationship between several circadian locomotor parameters and mood related behaviors in rats. In particular there is an association between activity during the light, under entrained conditions as well as LL, and anxiety and depression-like behaviors. We speculate that individual differences in the responsiveness to light could be mediating these responses. Consistent with this type of association, it has been shown that mice selectively bred for high anxiety- and depression-like behaviors are less responsive the phase shifting effects of light [17]. An alternative explanation would be that light is directly influencing mood independently of the circadian system, as aberrant light schedules have been shown to alter mood related behaviors in mice [39]. Bright light therapy is an effective treatment for individuals with either seasonal or non-seasonal depression [40]. The circadian phase of bright light therapy may be important for the antidepressant effects. Light therapy is frequently prescribed in the morning [41]; however, evening treatments have also been shown to be effective [42], suggesting that there may be some “non-circadian” pathways mediating these effects.

The current study contributes to our understanding of the relationship between circadian locomotor rhythms and mood-related behaviors. One of the limitations to this study is that all of the subjects were exposed to multiple lighting conditions prior to behavior testing. Exposure to different light cycles may produce aftereffects [36], but these are not usually observed after constant dark or constant light schedules. To minimize potential aftereffects, rats were given two weeks of 12h:12h LD between manipulations. The choice to use an inbred rat strain allowed us to control for individual differences in locomotor and mood-related behavior due to genetic variability. Although individual differences in circadian locomotor parameters are determined in part by genetics, there are other variables that can affect an individual’s rhythms, including, social interactions, exercise, age and environmental lighting [43–46]. To account for the influence of social interactions we single housed the rats, to control for the effect of age all rats were young, and environmental lighting was standardized between boxes. Our choice to minimize the number of extraneous variables influencing circadian rhythms, particularly the use of an inbred strain may limit the degree of inter-subject variability and thereby reduce the number of correlations we observed. Lastly, although rats were housed under 12h:12h LD conditions at multiple points during the study we chose to focus on the final 12h:12h LD cycle. It is possible that circadian parameters fluctuate over time, but this does not appear to be the case as we found a significant positive correlation between circadian locomotor activity between entrained conditions pre and post light manipulations. This suggests that individual differences in these circadian parameters are stable over time, which is consistent with previous findings [37]. Therefore, we are confident that the results from this time point reflect the general relationship between circadian locomotor parameters and mood-related behaviors.

## Conclusion

Disrupted circadian rhythms are a cardinal feature of many psychiatric conditions, and there is accumulating evidence to suggest that disrupted circadian rhythms are involved in the pathogenesis of some of these disorders. We demonstrate here that individual differences in circadian locomotor parameters are associated with depression- and anxiety-like behaviors in Lewis rats. With a more comprehensive understanding of the relationship between individual differences in circadian rhythms and mood there is potential to identify novel biomarkers that can be used to identify individuals at risk for developing certain psychiatric conditions.
